# Unmet needs in sexual health in bladder cancer patients: a systematic review of the evidence

**DOI:** 10.1186/s12894-020-00634-1

**Published:** 2020-06-03

**Authors:** Agustina Bessa, Rebecca Martin, Christel Häggström, Deborah Enting, Suzanne Amery, Muhammad Shamim Khan, Fidelma Cahill, Harriet Wylie, Samantha Broadhead, Kathryn Chatterton, Sachin Malde, Rajesh Nair, Ramesh Thurairaja, Pardeep Kumar, Anna Haire, Saran Green, Margaret Northover, Karen Briggs, Mieke Van Hemelrijck

**Affiliations:** 1 King’s College London, School of Cancer and Pharmaceutical Studies, Translational Oncology & Urology Research (TOUR), TOUR, Guy’s Hospital, 3rd Floor Bermondsey Wing, London, SE1 9RT UK; 2 The Royal Marsden, Department Urology, London, UK; 3 Department of Surgical Sciences, Uppsala University, Uppsala, Sweden; 4 Department of Biobank Research, Umeå University, Umeå, Sweden; 5 Department of Oncology, Guy’s and St Thomas’ NHS Foundation Trust, London, UK; 6 Department of Urology, Guy’s and St Thomas’ NHS Foundation Trust, London, UK

**Keywords:** Bladder cancer, Sexual health, Radical cystectomy, Health-related quality of life, Mental wellbeing

## Abstract

**Background:**

Bladder cancer (BC) treatment can have a detrimental effect on the sexual organs of patients and yet assessment of sexual health needs has been greatly overlooked for these patients compared to those who have undergone other cancer therapies.

**Methods:**

This review was conducted in accordance with the Preferred Reporting Items for Systematic Reviews and Meta-analyses (PRISMA) guidelines in July 2019. Studies were identified by conducting searches for Medline (using the PubMed interface), the Cochrane Central Register of Controlled Trials (CENTRAL) and Ovid Gateway (Embase and Ovid) using a list of defined search terms.

**Results:**

15 out of 37 studies included men only, 10 studies women only and 11 both sexes. Most participants were aged 50 to 65 years. Most studies (*n* = 34) focused on muscle invasive BC and only three on non-muscle invasive BC. Measurements of sexual dysfunction, including erection, ejaculation, firmness and desire, were the most commonly used measurements to report sexual health in men. In women, lubrification/dryness, desire, orgasm and dyspareunia were the most commonly reported. Twenty-one studies evaluated sexual dysfunction based on validated questionnaires, two with a non-validated questionnaire and through interviewing participants.

**Conclusion:**

While recognition of the importance of the inclusion of psychometric measurements to assess sexual health is growing, there is a lack of consistent measures to assess sexual health in BC. With the focus on QoL arising in cancer survivorship, further studies are needed to develop, standardize and implement use of sexual health questionnaires with appropriate psychometrics and social measures to evaluate QoL in BC patients.

**Trial registration:**

“PROSPERO does not currently accept registrations for scoping reviews, literature reviews or mapping reviews. PROSPERO is therefore unable to accept your application or provide a registration number. This decision should not stop you from submitting your project for publication to a journal.”

## Background

It is well known that the diagnosis and treatment of cancer causes significant physical, psychological, and social effects that interfere with a person’s sexual health. Indeed, it has been estimated that between 40 and 100% of cancer patients will experience a degree of sexual dysfunction [1]. Sexual dysfunction is characterized by disturbances in sexual desire and in the psychophysiological changes associated with the sexual response cycle in men and women or pain during intercourse [2, 3]. The degree to which sexual dysfunction has been studied in different cancer types varies, with most studies performed in patients with prostate, breast or gynaecological cancers. The World Health Organization (WHO) defines sexual health as a state of physical, emotional, mental, and social wellbeing related to sexuality, and is not merely the absence of disease, dysfunction or infirmity [4]. Therefore, sexual health has to be evaluated holistically due to the complex interactions between biological, psychological, interpersonal and social/cultural factors [5], as all these factors may effect sexual function and wellbeing [6].

Bladder cancer (BC) treatments are known to have a potential detrimental effect on both the genitals and the internal sexual organs of patients and yet it has been greatly overlooked compared to other cancer therapies [7]. Research of the treatment impact on sexual health has been widely identified as an unmet need in bladder cancer patients [8, 9].

Non-muscle invasive bladder cancer (NMIBC) accounts for about 70% of all bladder cancers [10] and those are mostly treated with transurethral resection of the bladder tumor (TURBT), followed by intravesical chemotherapy (22%) or immunotherapy with bacillus CalmetteGuerin (BCG, 29%) [11]. Muscle invasive bladder cancer (MIBC) are commonly treated with cystectomy [11, 12]. The vast majority of studies currently available for bladder cancer focus on those who have undergone a radical cystectomy. Among those, many studies evaluate sexual-sparing radical cystectomy with the goal of reaching definitive oncologic control while attempting preservation of sexual function [13].

Current European Association of Urology (EAU 2019) guidelines [14] recommend that a standard radical cystectomy in men should include removal of the bladder, prostate, seminal vesical, distal ureters and regional lymph nodes, while in women removal of the bladder, entire urethra and adjacent vagina, uterus, distal ureters and regional lymph nodes is recommended. The guidelines also stated that a prostate-sparing radical cystectomy could be considered in carefully selected patients if oncologically safe. However, a sexual organ preserving operation in women is not advised, largely due to a paucity of available evidence [14]. Such treatments can induce serious complications and greatly impact the patient’s body image and quality of life (QoL) [11]. Health-related QoL (HRQoL) can be formally defined as the extent to which one’s usual or expected physical, emotional, and social wellbeing are affected by a medical condition or its treatment [15]. HRQoL issues of special concern to patients with BC include, among others, threats to body image and sexuality [16, 17].

With the focus on HRQoL arising in cancer survivorship, it is needed to closely address and evaluate post-treatment sexual dysfunction and offer goal-directed treatment. To date no detailed assessment of the overall burden of sexual health in bladder cancer patients has been made. This systematic literature review aimed to provide a consolidated overview of studies that address sexual health in bladder cancer patients. We specifically aimed to assess the methodology used to evaluate sexual health in terms of coverage and validation after BC treatment, which may provide a basis for future studies.

## Methods

This review was conducted in accordance with the Preferred Reporting Items for Systematic Reviews and Meta-analyses (PRISMA) guidelines in July 2019. A detailed overview of the protocol is provided in Appendix 1.

### Search strategy and inclusion criteria

Studies were identified by conducting searches for Medline (using the PubMed interface), the Cochrane Central Register of Controlled Trials (CENTRAL) and Ovid Gateway (Embase and Ovid) between May and July 2019 using a list of defined search terms (see Appendix 2). To be included in the analysis, the studies must have met the following criteria: study on the impact of bladder cancer treatment on sexual health/dysfunction and reported outcomes specifically for sexual health/dysfunction (e.g. erection, ejaculation, dryness).

### Data collection and analysis

Initially, the titles of the studies were screened to identify the relevant studies. The abstracts and subsequently full texts were then carefully read to identify those which met the inclusion criteria. Two independent reviewers (AB and RM) used the exact same search strategy and inclusion criteria and after each screening phase, a discussion was conducted with a third party (MVH) to match and decide on the included studies. Information on patient characteristics, number of study participants and type of treatment, as well as sexual health outcomes and mental wellbeing information was extracted from each study. The latter data was collected as sexual dysfunction may also have an impact on HRQoL through effects on mental wellbeing (4). The references of the included studies were also reviewed to ensure no relevant citation was missed.

## Results

### Quantity of evidence identified

**Fig. 1:**
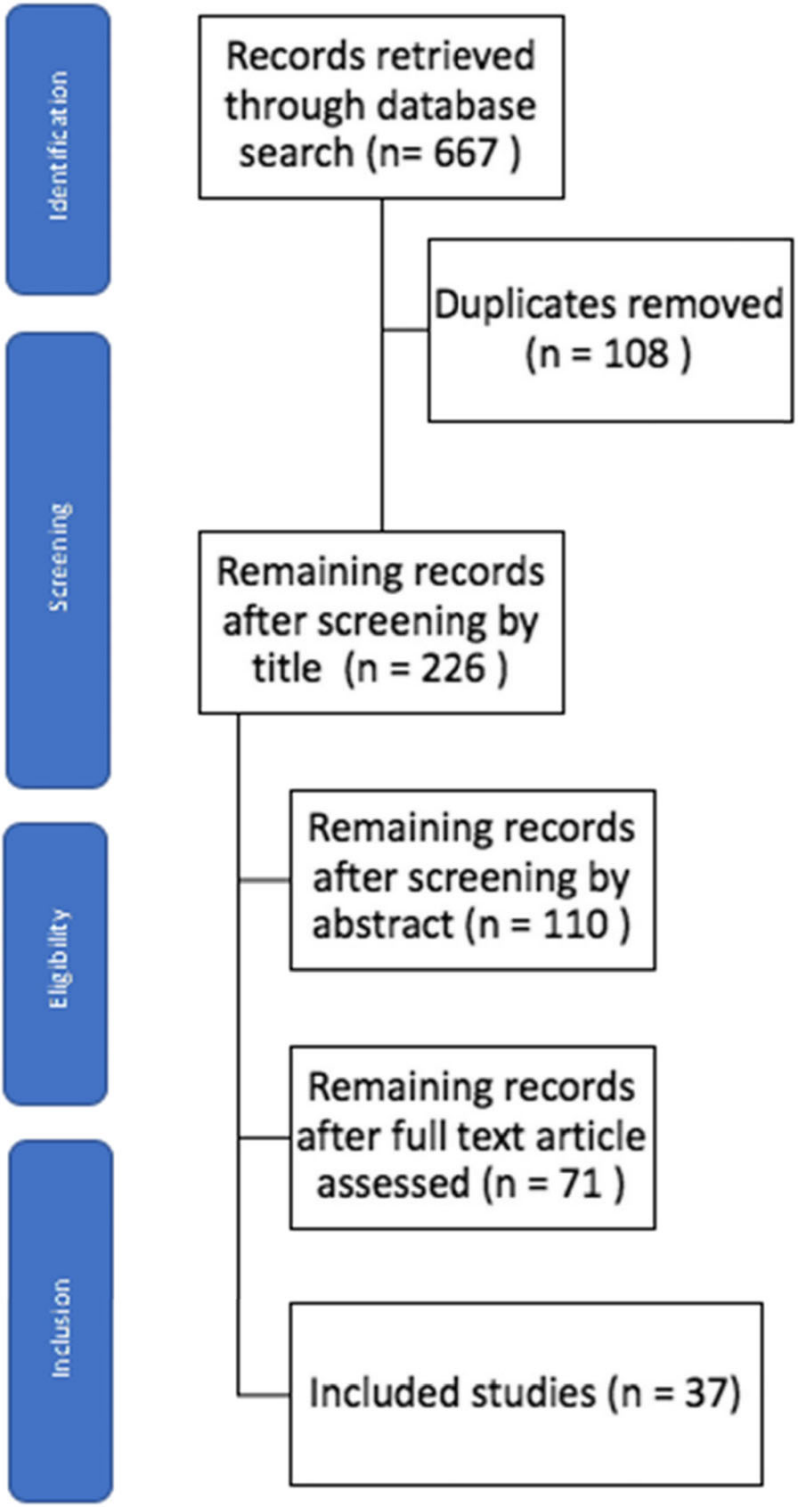
PRISMA Flow diagram for selection of studies in systematic review

The selection process for records to be included in the review was carried out according to PRISMA protocol, and this is demonstrated in a PRISMA flowchart in Fig. 1. A total of 667 records were collected from the literature search and 108 duplicates were removed. All titles were initially screened and 226 remained for abstracts screening. Of those, 71 remained for full text analysis. After the full text was read, 37 studies matched the inclusion and exclusion criteria and were included in this systematic review (Table 1, Appendix 3).

**Table 1:** Characteristics of the patient profile included in the studies

Patient profile	Studies (n)
**Sex (%)**	
Only men	15 (39.5%)
Only women	10 (28.9%)
Both sex	12 (32.4%)
**Age**	
26–49	5 (13.5%)
50–65	28 (75.6%) ^a^
66–80	4 (10.8%)
81–95	0
**Disease stage (%)**	
MIBC	34 (91.9%)
NMIBC	3 (8.10%)

^a^One study does not provide the mean age but state that patients were between 56 and 75 years old

### Characteristics of the patient profiles

Of the 37 studies included in the systematic review, 15 studies (40.5%) included men only, 10 studies (27.0%), women only and 12 studies (32.4%) included both sexes. The exact number of women and men in the studies that included both sexes is not clearly described for all studies. With respect to age, in 28 studies (75.6%) participants were aged between 50 to 65 years, in five studies (13.5%) 26 to 49 years, in four studies (10.8%) 66 to 80 years and none included participants above 80 years old. The vast majority of studies [*n* = 34 (91.9%)] included MIBC and only three (8.10%) focused on NMIBC. Patient profiles for each study are described in Table 1.

### Measurement of sexual health by sex

Measurements of sexual dysfunction, including erection (73.0%), ejaculation (35.1%), firmness (8.10%) and desire (18.9%), were the most commonly used measurements to report sexual health in men. In women, lubrification/dryness (27.0%), desire (24.3%), orgasm (27.0%) and dyspareunia (29.7%) were the most commonly reported (Table 2).

**Table 2:** Measures of reported sexual dysfunction by sex

Sex	Measurement	Number of Studies (%)
**Men**	Erection	27 (73.0%)
	Firmness	3 (8.10%)
	Desire	7 (18.9%)
	Ejaculation	13 (35.1%)
**Women**	Lubrification / Dryness	10 (27.0%)
	Desire	9 (24.3%)
	Orgasm	10 (27.0%)
	Dyspareunia	11 (29.7%)

### Comparison of sexual dysfunction outcome by treatments and measurements

Thirty-three (89.2%) studies addressed sexual dysfunction following radical cystectomy, one (2.7%) following radical radiotherapy, one (2.7%) after cystoscopy, and two (5.4%) after other treatments for bladder cancer. There are no studies looking at TURBT only. In four studies (10.8%), participants were treated with drug therapy for their sexual dysfunction. Twenty-one studies (56.8%) evaluated sexual dysfunction based on validated questionnaires, two (5.4%) with a non-validated questionnaire and 14 (37.8%) through interviewing participants.

### Patient and partners sexual satisfaction and mental wellbeing

Seven studies evaluated patient sexual satisfaction and mental wellbeing (Table 3). None of those included both patient and partner satisfaction and mental wellbeing. One study included sexual satisfaction for both patients and partners, but did not report on mental wellbeing [18]. Regarding mental wellbeing, perception of body image [20, 22, 24], worry about daily life [19], feeling of anxiety [23] and depression [22] were the most commonly reported outcomes. Six of those studies included patients with MIBC and one NMIBC. Six studies included patients in the age range of 50–65 years and two included patients 66–80 years old. Overall, radical cystectomy was found to be associated with negative feelings regarding patient’s body image and consequently a negative effect in sexual satisfaction. Patients who underwent a bladder sparing technique had a better perception of their body and a better sexual satisfaction. There is no consistency in the measurements used for sexual satisfaction and/or mental wellbeing: interviews and semi-structured interviews, sexual function index questionnaire, QoL questionnaire (SHIM and IFSF) and a functional assessment of cancer therapy questionnaire.

**Table 3:** Patient and partner sexual satisfaction and mental wellbeing

Study [ref]	Age range	Self-reported patient and partner satisfaction (if data available)	Mental wellbeing	Measurement
[18]	50–65	Participants were found to have frequent erectile dysfunction, ejaculation disorder, fear of hurting spouse/partner and leakage during sexual intercourse. The participants’ spouse/partner were found to have fear of hurting, decrease in sexual desire, inability to get pleasure/orgasm and avoiding sexual intercourse.	No data.	Interview
[19]	50–65	20% of participants were not sexually satisfied. No data regarding partner.	Among the patients, 42 (84%) of them were not feeling bad about their bladder tumors and 37 (74%) were not worrying about their daily lives. Moreover, 12 (24%) patients were not interested with sexuality.	International Sexual Function Index (IIEF-5) and Female Sexual Function Index (FSFI
[20]	50–65	TMT was associated with higher sexual quality of life. No data regarding partner.	Compared with RC, the patients treated with TMT reported significantly better perception of their body image. Patients who underwent TMT had significantly fewer concerns about their appearance and less life interference from their cancer diagnosis. Overall, the negative impact of cancer was significantly less for patients who had undergone TMT.	QoL questionnaire
[21]	66–80	37.5% patients in the study agreed that treatment affected their sexual function. No data regarding partner.	At the time of diagnosis, patients of both sexes reported relatively high feelings of depression.	Semi-structured interviews.
[22]	66–80	Forty-six patients enrolled in the study. Of those, only 27 patients responded to the statement “I am satisfied with my sex life”. Approximately the same number of patients agreed as disagreed, but the main response was neither to agree nor to disagree with this statement. Thirty-one patients responded to the statement “My sex life is not functioning”. Most patients agreed with this statement. Only 23 patients responded to the statement “I am very happy with my sex life”. Again, most patients neither agreed nor disagreed. No data regarding partner.	The patients were asked whether the change in their body appearance had resulted in psychological problems which had influenced their sex life. Twenty-five patients (61%) answered that this was not the case, whereas 11 (27%) answered that this was the case.	Female Sexual Function Index (FSFI) questionnaire
[23]	50–65	Radical cystectomy group, through the 4 measurements points, started, stayed and ended on a lower level than the case-control patients regarding overall sexual satisfaction. No data regarding partner.	The mean score for trait anxiety was similar in both groups (RC and CC). Only 5.6% of all RC patients had a high anxious personality compared to 20% in the CC group, but this was not statistically significant.	International Index of Erectile Function (FSFI) and International Index of Erectile Function (IIEF)
[24]	50–65	No data regarding sexual satisfaction. No data regarding partner.	Among patients who had undergone RC, 8.5% of the responders reported being unhappy with their body image, compared with the 5% reported by patients in BI group.	FACT-BL + additional concerns

## Discussion

Sexual dysfunction is a common phenomenon after bladder cancer treatment and yet sexual health is frequently overlooked. Of the 37 included studies included in our review, 15 studies included men only, 10 studies included women only and 12 studies included both sexes. Most studies included participants aged between 50 to 65 years. The majority of studies focused on patients with MIBC. The main outcomes measured in men were erection, ejaculation and desire and in women, lubrification/dryness, orgasm and dyspareunia. A broad range of measures were used in the studies including validated patient reported outcome tools, such as the SHIM and the IFSF questionnaire and interview-based studies.

One of the main observations following our systematic review is the heterogeneity in measurements used for sexual health. The SHIM tool is commonly used in prostate cancer and has also been most commonly used in the studies identified here. In female patients the available measurement tools are broader and less commonly implemented in practice. The most commonly used tool we found was the IFSF questionnaire. Both tools have been criticised for their lack of psychometric measures [25]. Indeed, the need for a robust measure of sexual function in bladder cancer that includes psychometric assessment is well recognised, but not yet developed [26]. Mohamed et al. (2014) identified unmet needs in MIBC patients and sexual function was a significant concern [8]. With no evidence of consistent clinical measures from practice, these findings are unsurprising. Patient and public involvement is needed to develop and include appropriate psychometrics measures in sexual health questionnaires. As previously noted by Peter Selby and Galina Velikova [27], patient input is essential, but the process is time consuming, sometimes quite expensive and can involve large numbers of patients or the general population to achieve meaningful results. Therefore, individuals and groups have tended to develop their own questionnaires which led to a lack of general consensus in the field and a confusing number of questionnaires.

Moreover, our systematic review highlights how sexual health may negatively impact on mental wellbeing and HRQoL – and hence indicates the need to address this gap for patients. As suggested by the definition of the WHO [4], sexual health has to be evaluated from a psychosocial perspective and not only the physical and physiological aspects. Although erectile dysfunction in men and physical and physiologic changes associated with cystectomy in women are the dominant factor driving sexual function, other causes of sexual dysfunction, such as depression or anxiety related to changed body image, distress regarding partner reaction to an altered body as well as the degree of problems that patients are experiencing due to their sexual function (bother), should be evaluated and managed.

Currently, HRQOL questionnaires like the Functional Assessment of Cancer Therapy – Bladder Cancer (FACT-BC) do include questions about interest in sex, while the Bladder Cancer Index (BCI) also includes questions about desire, arousal, sensation, and orgasm. Several EORTC questionnaires include a limited number of sexual functioning items. However, there is no single self-reported measure that covers the entire range of sexual health. Recently, the European Organization on Research and Treatment of Cancer (EORTC) developed an EORTC Sexual Health Questionnaire (EORTC SHQ-22) for assessing sexual health in cancer patients [28]. In addition to that, HRQOL assessment should target patient’s current or very recent HRQOL status. This is of interest as (1) it has been suggested that patients overestimate their baseline HRQOL in the sexual function domains by 27% [29]; and (2) it would allow to better understand the degree of problems experienced by patients (measuring “bother”). It is also important to note that function and bother do not necessarily correlate as demonstrated by Letwin et al. [30] and that despite sexual health issues being an important concern to patients, they often experience difficulties in disclosing their complaints with health care providers or their partners. Lastly, as sexual wellbeing is closely correlated with social interactions, patients’ relationship status should also be considered when evaluating sexual wellbeing and its impact on HRQOL.

In our systematic review, most studies addressed sexual health in patients with MIBC who underwent radical cystectomy and only four studies focused on patients with NMIBC. This is potentially affecting the generalisibility of our observations as it is important to note that treatments for NMIBC and MIBC substantially differ, e.g. TURBT vs cystectomy. In a prospective study by Yoshimura et al. [31] on the impact of TURBT on QoL in patients with NMIBC, physical problems were found with the first TURBT, but when the fourth TURBT was performed, patients appeared to have adapted to frequent operations, although their general QoL remained affected. For cystectomy patients, it has been shown that although relationships with friends were unchanged, relationships with spouse or partner were disturbed by sexual problems [32]. Partner response to the presence of an external appliance, such as stoma, may also strain intimate relationships and contribute to a dysfunctional sex life. Nevertheless, these observations highlight that 1) the threat of recurrence, multiple cystoscopies, TURBT, and intravesical instillations make QoL assessment in superficial bladder cancer challenging; 2) the importance of looking at sexual function in patients with bladder cancer in a broader way is needed, i.e. including both the physical perspective as well as the patients’ partner and relationships.

There are limitations of this study that warrant further discussion. The analysis of the included studies was unable to provide an extensive coverage of sexual health in NMIBC patients for which the impact of treatment in sexual health and consequently QoL may also be significative. Due to the nature of the methods used to measure sexual health and the lack of psychometric measures, further research is needed to better understand the role of bladder cancer treatment and psychosocial factors of sexual functioning.

## Conclusion

While recognition of the importance of the inclusion of psychometric measurements to assess sexual health is growing, this systematic review highlights the lack of consistent measures to assess sexual health in bladder cancer patients. With the focus on HRQoL arising in cancer survivorship, further studies are needed to develop, standardize and implement the use of sexual health questionnaires with appropriate psychometrics and social measures for bladder cancer patients.

## Data Availability

Not applicable.

## Appendix 1

**PROTOCOL:** Systematic literature review on sexual health for patients with bladder cancer.

**AIM:** To consolidate an overview of studies that address sexual health in bladder cancer patients

**Objectives**

- Assess the methodology used to evaluate sexual health in terms of coverage and validation after bladder cancer treatment,
- Understand the role of bladder cancer treatment and psychosocial factors of sexual functioning

**METHODS**

Reports of current systematic review protocol adhere to the Preferred Items for Systematic Reviews and Meta-Analysis Protocol (PRISMA-P) checklist. The systematic review itself will adhere to the PRISMA.

**Criteria for considering studies for this review.**

**Types of studies.**

Any study that investigated sexual function or sexual dysfunction; any study where sexual function/sexual dysfunction/sexual health were measured by self-reports or an interview. Studies were original full text report in English and were published in peer-reviewed journals.

**Types of participants.**

The population of interest will be bladder cancer patients. Demographic factors are no exclusion criteria except for age (< 18 years of age).

**Types of outcomes measures.**

For both primary and secondary outcome measures, there will be no exclusion based on length of follow-up.

**Primary outcomes.**

Sexual function or sexual dysfunction in bladder cancer patients.

**Secondary outcomes.**

Treatment and psychosocial factors of sexual functioning.

**SEARCH METHODS FOR IDENTIFICATION OF STUDIES.**

**Electronic searches.**

The following electronic databases will be searched from inception until the search date: the Cochrane Central Register of Controlled Trials (CENTRAL), MEDLINE (using the PubMed interface), Embase (using the embase.com interface) and PsychINFO.

**Data collection and analysis.**

**Selection of studies.**

All references found through the search process will be downloaded in a database created by reference management software (Endnote). After removing duplicates in Endnote, all references will be imported into Covidence for screening purposes. Obviously irrelevant studies, based on title and abstract, will independently be excluded by two review authors. After screening the titles and abstracts, two review authors will independently assess full-text reports for eligibility. Discrepancies will be discussed with a third review author. Reasons for exclusion of full-texts will be documented. Studies will be excluded if no full-text is available. Abstracts in any other language than English will be excluded. There will be no language restriction for full texts, and translations will be carried out if necessary. If studies have multiple publications with the same outcome(s) reported, manuscripts with the longest follow-up will be selected for inclusion.

**Data extraction.**

For each included study in the review, at least the following information will be extracted:

- General information: report title, report ID, authors’ names, country, contact address, language of publication and year of publication.

- Population and setting: population description (from which study participants are drawn), setting when applicable (eg, inpatient, outpatient, hospital setting, home setting and combination) and inclusion and exclusion criteria.

- Methods: aim of study, design, start and end date and duration of participation.

- Participants: number of participants, received treatment.

- Outcomes: outcome name, time points measured and reported.

- Results: measurement of sexual function and sexual dysfunction, psychosocial factors measured.

**Dealing with missing data.**

If essential data are not available in the publication, we will first attempt to contact the study authors. If this is not possible, we will try to back-calculate from the data presented. If data will be obtained from other study authors, this will be reported in the review in a transparent manner. This way, we can keep in mind that these missing data obtained from study authors were not peer reviewed. Studies assessing lifestyle interventions may have issues with compliance. Therefore, reasons for missing data (eg, dropouts, losses to follow-up and withdrawals) will be carefully reported.

**Data synthesis.**

The findings from the included studies will be summarised in a table format.

**ETHICS AND DISSEMINATION.**

Ethics approval is not required, as no primary data will be collected. Results will be disseminated through a peer-reviewed publication.

The results of this systematic review could also have potential limitations in terms of biased results due to the nature of the patient reported outcomes.

## Appendix 2

**Search strategy**

PubMed.

“Urinary Bladder Neoplasms”[MESH] OR “Urinary Bladder/abnormalities”[MESH] OR “Urinary bladder/surgery”[MESH] OR “Carcinoma, Transitional Cell”[Mesh] OR “bladder cancer”[TIAB] OR “bladder cancers”[TIAB] OR “bladder carcinoma”[TIAB] OR “bladder carcinomas”[TIAB] OR “bladder malignancy”[TIAB] OR “bladder malignancies”[TIAB] OR “bladder neoplasm”[TIAB] OR “bladder neoplasms”[TIAB] OR “bladder tumor”[TIAB] OR “bladder tumors”[TIAB] OR “bladder tumour”[TIAB] OR “bladder tumours”[TIAB] OR “transitional cell carcinoma”[TIAB] OR “transitional cell carcinomas”[TIAB] OR “transitional cell cancer”[TIAB] OR “transitional cell cancers”[TIAB] OR “transitional cell malignancies”[TIAB] OR “transitional cell tumor”[TIAB] OR “transitional cell tumors”[TIAB] OR “transitional cell tumour”[TIAB] OR “transitional cell tumours”[TIAB]

### And

“Sexual function” OR “sexual dysfunction” OR “wellbeing” OR “intimacy” OR “sexuality” OR “sexual physiology” OR “sexual physiopathology” “sexual heath”

### And

“Cystectomy” OR “radical cystectomy” OR “partial cystectomy” OR “TURBT” OR “Transurethral resection of bladder tumour” OR “BCG” OR “chemotherapy” OR “Radiotherapy” OR “Bacillus Calmette–Guérin vaccine”.

Cochrane Library.

“Urinary Bladder Neoplasms” OR “Urinary Bladder abnormalities” OR “Urinary bladder surgery” OR “Carcinoma, Transitional Cell” OR “bladder cancer” OR “bladder cancers” OR “bladder carcinoma” OR “bladder carcinomas” OR “bladder malignancy” OR “bladder malignancies” OR “bladder neoplasm” OR “bladder neoplasms” OR “bladder tumor” OR “bladder tumors” OR “bladder tumour” OR “bladder tumours” OR “transitional cell carcinoma” OR “transitional cell carcinomas” OR “transitional cell cancer” OR “transitional cell cancers” OR “transitional cell malignancies” OR “transitional cell tumor” OR “transitional cell tumors” OR “transitional cell tumour” OR “transitional cell tumours”

### And

“Sexual function” OR “sexual dysfunction” OR “wellbeing” OR “intimacy” OR “sexuality” OR “sexual physiology” OR “sexual physiopathology”

### And

“Cystectomy” OR “radical cystectomy” OR “partial cystectomy” OR “TURBT” OR “Transurethral resection of bladder tumour” OR “BCG” OR “chemotherapy” OR “Radiotherapy” OR “Bacillus Calmette–Guérin vaccine”.

Embase.

Urinary Bladder Neoplasms OR Urinary Bladder abnormalities OR Urinary bladder surgery OR Carcinoma, Transitional Cell OR bladder cancer OR bladder cancers OR bladder carcinoma OR bladder carcinomas OR bladder malignancy OR bladder malignancies OR bladder neoplasm OR bladder neoplasms OR bladder tumor OR bladder tumors OR bladder tumour OR bladder tumours OR transitional cell carcinoma OR transitional cell carcinomas OR transitional cell cancer OR transitional cell cancers OR transitional cell malignancies OR transitional cell tumor OR transitional cell tumors OR transitional cell tumour OR transitional cell tumours

### And

Sexual function OR sexual dysfunction OR sexual health OR wellbeing OR intimacy OR sexuality OR sexual physiology OR sexual physiopathology

### And

Cystectomy OR radical cystectomy OR partial cystectomy OR TURBT OR Transurethral resection of bladder tumour OR BCG OR chemotherapy OR Radiotherapy OR Bacillus Calmette Guerin vaccine OR Intravesical therapy OR Systemic immunotherapy.

## Appendix 3

**Table 4:** Treatment, measurement and outcome

Study number [ref]	Patient profile	Treatment, preserving technique and/or urinary diversion	Measurement	Sexual function outcome
1 [33]	Group 1 comprised 58 subjects (mean age 65 years) Group 2 comprised 50 subjects (mean age 61 years) Group 3 comprised 54 healthy subjects (mean age 63 years	Radical cystectomy compared in ileal conduit diversion, modified S-pouch and healthy subjects	Interviews	Erectile dysfunction was worse in groups 1 (who received ileal conduit diversion) (29%) and 2 (modified S-pouch neobladder) (35.5%) compared to group 3 (healthy subjects) (83.2%). The quality of erection (firmness) was similarly affected in groups 1 (17.7%) and 2 (22.2%) compared with group 3 (83.3%) Sexual dysfunction was also evaluated for women and was not pronounced as in the men. Sexual desire was equally present in all groups and patients in group 1 expressed higher levels of dissatisfaction than the other two groups.
2 [34]	Twenty men in each group (mean age 58 and 59 years)	Radical cystectomy with prostate capsule sparing or nerve sparing and neobladder urinary diversion	Sexual Health Inventory for Men questionnaire (SHIM)	Compared to baseline, sexual function decreased similarly for both groups. 55 and 65% of prostate capsule sparing and nerve sparing patients used aids or medications for erectile function, respectively.
3 [18]	Twenty patients and their spouses/partners (mean age 59 and 58, respectively)	Cystectomy followed by urostomy	Interviews	Participants were found to have frequent erectile dysfunction, ejaculation disorder, fear of hurting spouse/partner and leakage during sexual intercourse. The participants’ spouse/partner were found to have fear of hurting, decrease in sexual desire, inability to get pleasure/orgasm and avoiding sexual intercourse.
4 [35]	Ninety-one patients and 59 were included in the continent and incontinent group, respectively (mean age 55 and 59.5 years, respectively)	Radical cystectomy with orthopaedic neobladder or uretro-sigmoidostomy (continent group) or uretero-cutanoustomy or had ileal conduit (incontinent group)	Sexual Health Inventory for Men Questionnaire (SHIM)	Regarding sexual function, from 121 patients, 29 (23.9%) underwent nerve-sparing and 59 (48.7%) non nerve- sparing procedure. Erectile dysfunction affected 78.4% of them. There was a significant difference between both groups with lower degrees of erectile dysfunction in nerve-sparing group. After one year postoperatively, 19/29 (65.5%) patients who underwent nerve-sparing radical cystectomy demonstrated spontaneous complete tumescence, 6 (20.7%) achieved erections with the use of oral PDE5 inhibitors while 4 (13.8%) required intra-cavernosal injection therapy. All patients treated with non-nerve sparing RC required intra-cavernosal injection therapy to achieve erections.
5 [36]	Hundred eighty-five men were included (mean age of 57 years)	Prostate sparing cystectomy	Interviews	In total, 136 patients (86.1%) maintained erectile function, of whom 27 (19.9%) successfully used sildenafil, and 6 patients (4.4%) used intra-cavernous injections.
6 [19]	Forty-four men and 6 women (mean age 57.6 years)	Cystoscopy	International Sexual Function Index (IIEF-5) and Female Sexual Function Index (FSFI)	Mild-moderate erectile dysfunction was found in men and sexual dysfunction in women.
7 [37]	Eleven men underwent conventional radical cystoprostatectomy and 14 patients had prostate-sparing cystectomy (mean age 61.55 and 57.5 years, respectively)	Orthotopic neobladder reconstruction after prostate-sparing cystectomy and conventional radical cystoprostatectomy	International Index of Erectile Function-5 (IIEF-5)	After surgery, IIEF-5 scores in conventional radical cystoprostatectomy and prostate-sparing cystectomy groups were 3.7 and 16.0, respectively.
8 [38]	Sixty-three men and 4 women (mean age 63 years)	Robot-assisted radical cystoprostatectomy	Interview	With regard to sexual potency, twenty (43%) patients with neobladder reported spontaneous erections prior to surgery, 14 (29%) erectile dysfunction of some degree, and in 13 (28%) it is unknown. Of the 20 patients with normal sexual function prior to surgery, 90% had spontaneous erections that allowed them to have satisfactory sexual relations; of them, 3 managed it with the help of IPDE-5. Of the 14 patients who already reported some prior erectile dysfunction, 9 achieved satisfactory erections with IPD5.
9 [20]	Hundred and nine patients underwent radical cystectomy and 64 patients underwent trimodality therapy (mean age 66 and 65, respectively)	Radical cystectomy and bladder-sparing trimodality therapy	QoL questionnaire	Male patients who had received trimodality therapy reported less difficulty gaining or maintaining an erection and decreased ejaculation problems compared with those who had undergone radical cystectomy. Patients who had undergone trimodality therapy also reported being less uncomfortable with being sexually intimate and less worried about contaminating their partner during sexual contact.
10 [21]	Twenty-two men and 8 women (mean age 67 years)	Radical cystectomy and urinary diversion	Semi-structured interviews	Overall, 25 and 18.18% of younger and older patients, respectively, reported a discussion with the physician regarding changes in sexual function. Following surgery, younger patients were more likely to report problems with sexual dysfunction.
11 [39]	Women between 20 and 28 years (mean 24 years) were 30.77%. Women of age range of 32–37 (mean 35 years) were 23.07% Women of age range of 40–54 (mean 49 years) were 48.5%	Radical cystectomy with preservation of genital organs and orthotopic ileal neobladder urinary diversion	Female Sexual Function Index (FSFI) questionnaire	Women who had reported good sexual function had DSFI score ranging from 20 to 32, which was reported by 84.61%. One woman had a score of 15, while another woman was not married and had no partner and she reported a score of zero.
12 [22]	Seventy-one women (mean age of 67 years).	Radical cystectomy	Female Sexual Function Index (FSFI) questionnaire	Only 37% of the patients were sexually active post-surgery and those who were still sexually active tended to have less frequent sexual activity. A comparison of sexual desire before and after surgery found that 15 patients (37%) reported that this was unchanged, 22 patients (54%) that their desire for sex had decreased and one person that it had increased. Regarding ability to reach orgasm before and after surgery, 16 patients (39%) reported that this was unchanged and 18 (44%) that it was now harder to reach orgasm, while no one reported that it was easier to reach orgasm.
13 [40]	Sixty-eight men and 25 women (mean age 65 years)	Bacillus Calmette–Guérin (BCG) treatment	Semi-structured qualitative interviews	For those individuals who were sexually active, 60.0% of males experienced difficulty gaining or maintaining an erection and 43.1% of males had problems with ejaculation. Among sexually-active females, 62.5% experienced problems including vaginal dryness. Nearly 50% of participants reported that it was very helpful to discuss concerns about sexual function with their partners.
14 [34]	Forty patients (mean age 58 years)	Prostate capsule sparing and nerve sparing cystectomies	Sexual Health Inventory for Men Questionnaire (SHIM)	Average sexual function at 12 months compared with baseline decreased by 1 +/− 11 and 23 +/− 30 points for prostate capsule sparing and nerve sparing, respectively. Of patients with prostate capsule sparing and nerve sparing cystectomy, 55 and 65%, respectively, used aids or medication for erectile function.
15 [23]	Eighteen patients undergoing radical cystectomy and 20 control (mean age 62.3 and 64.9 years, respectively)	Radical cystectomy	International Index of Erectile Function (FSFI) and International Index of Erectile Function (IIEF)	Regarding erectile dysfunction, intercourse satisfaction, orgasmic function, sexual desire and overall satisfaction—the radical cystectomy group started, stayed and ended on a lower level than the case control group throughout the study. No data reported for women.
16 [41]	Fifteen women patients (mean age of 42 years)	Internal genitalia sparing cystectomy	Female Sexual Function Index (FSFI)	Sexual intercourse was regained after 6 weeks, a duration which is significantly shorter than in women without genital preservation. The mean FSFI scores in the study group were significantly higher than in cystectomy women without genital sparing. Similarly, there were significant differences in favour of genital sparing cases in all domains of FSFI.
17 [42]	Seventy-eight men and 8 women (mean age 62 years)	Orthotopic neobladder	International Index of Erectile Function (IIEF-5) and visual analogue scale (VAS).	Most patients (88%) had a score of less than 10 points on IIEF-5, 5.3% of patients showed 10–14 points, 4% showed 15–19, and only 2.7% showed equal to or more than 20 points. Because evaluation by IIEF-5 had not been performed before surgery, we could not determine the precise influence of orthotopic neobladder on sexual function.
18 [43]	Four men (mean age of 45 years)	Radical cystectomy	Interview	All patients reported adequate sexual function with normal erections and satisfactory intercourse similar to that reported before surgery. The erectile activity was regained within the first 5 months after surgery. Two patients (50%) preserved antegrade ejaculation the other two patients reported failure of ejaculation.
19 [44]	Seven men (mean age of 40 years)	Radical cystectomy	Interview	Erectile function is normal in all patients with satisfactory sexual intercourse. Antegrade ejaculation was documented in six cases.
20 [45]	Four men and one woman (mean age 52.4 years)	Nerve sparing laparoscopic radical cystectomy	Female Sexual Function Index (FSFI) and International Index of Erectile Function score	Sexual function was preserved in the female patient and 2 of the 4 male patients.
21 [24]	Eighty-two patients who had undergone radical cystectomy and 177 other therapy (mean age 64.4 years)	Radical cystectomy and other therapy	FACT-BL + additional concerns	Patients with their bladders preserved had more interest in sex when compared with patients who had their bladders removed. Twenty-one percent of respondents who had their bladders preserved were not interested in sex as compared with 39% who underwent radical cystectomy. In the radical cystectomy group, about 89% of the respondents could not have and keep an erection, whereas in the bladder intact group only 32% of respondents could not have and keep an erection.
22 [46]	Thirteen women (mean age 56.7 years)	Nerve sparing cystectomy with urethra and vaginal sparing	Female Sexual Function Index (FSFI	All 6 patients were sexually active 1 year after surgery. There is a significant vaginal dryness, a lack of arousal, and dyspareunia often led to discontinuation of sexual intercourse in the non-nerve-sparing group, with only 1 patient sexually active.
23 [47]	Thirteen women (mean age 56.7 years)	Non nerve sparing and nerve sparing cystectomy	Female Sexual Function Index (FSFI	A reduction occurred in the mean score for desire, arousal, pain, and satisfaction. No change occurred in the mean score for orgasm or lubrication. One patient from the nerve-sparing group reported significant dyspareunia after cystectomy that had resolved. All 6 patients had resumed sexual activity at their 1-year follow-up visit.
24 [48]	Twenty-nine women (mean age of 61 years)	Cystectomy and orthotopic ileal neobladder	Female Sexual Function Index (FSFI)	The 11 of 17 patients who remained sexually active after cystectomy even had slight improvement. Six patients ceased to be sexually active postoperatively due to erectile dysfunction or partner death. One patient with interstitial cystitis became sexually active following cystectomy due to the loss of pelvic pain. Another 12 patients remained sexually inactive postoperatively.
25 (Nieuwenhuijzen, Meinhardt and Horenblas 2005)	Forty men (mean age of 56.9 years)	Prostate sparing cystectomy and neobladder	Interviews on erection and ejaculatory function and the international index of erectile function questionnaire	Erectile function could be determined in 40 patients, and potency was maintained in 77.5%, impaired in 12.5% and absent in 10%.
26 [49]	Forty-nine men (mean age 57.8 years).	Nerve-sparing radical cystectomy, orthotopic diversion, conduit diversion and cutaneous continent diversion	Sexual Health Inventory for Men (SHIM)	Total mean SHIM score decreased after radical cystectomy. Of the 49 patients, 42 (86%) did not have erections sufficient for vaginal penetration. Of these 42 patients, 22 (52%) tried sildenafil citrate. Of these 22 patients, only 2 (9%) responded positively. Only 9 (14%) of 49 sexually active men were potent after surgery. Of these 9 potent patients, 8 (89%) had undergone nerve-sparing radical cystectomy.
27 [50]	Twenty-eight patients (mean age of 51.0 years).	Supra-ampullar cystectomy	Self-administered questionnaire	Potency was preserved in 26 patients (92.8%), reporting satisfactory sexual intercourses; 15 patients (53.5%) also maintained antegrade ejaculation allowing procreation in 3 cases.
28 [51]	Twenty-seven women (mean age 54.79 years)	Radical cystectomy	Female Sexual Function Index (FSFI)	The total mean baseline Index of Female Sexual Function score decreased. The most common symptoms reported by the patients included diminished ability or inability to achieve orgasm in 12 (45%), decreased lubrication in 11 (41%), decreased sexual desire in 10 (37%), and dyspareunia in 6 patients (22%). Only 13 (48%) of the 27 patients were able to have successful vaginal intercourse, with 14 (52%) reporting decreased satisfaction in overall sexual life after radical cystectomy. Eight partners (30%) had a decrease in desire for sexual activity owing to apprehension after cancer diagnosis and treatment.
29 [52]	Ten men and 3 women (mean age 55 years)	Cystectomy and neobladder	Structured interview	Erectile function was insufficient in 3 men. In 5 men ejaculation was antegrade, in 4 it was retrograde and 1 had each experience from time to time. In the women lubrication and orgasmic feeling was reported to be normal after surgery.
30 [53]	Eighteen men (mean age 62.5 years)	Radical radiotherapy	Non-validated questionnaire	Eight out of 17 patients felt that their sex life was a lot worse since radiotherapy and four felt that their sex life was a little. Five patients felt that their sex life was unchanged. No patient felt their sex life had improved. Despite this, seven patients were unconcerned that their sex life had changed. Only eight of those 12 patients whose sex life had deteriorated were concerned about the decline in their sexual function and indeed only two noted great concern over the decline.
31 [54]	Hundred-twelve men (mean age 63 years)	Radical cystectomy	Semi-structured interview	Of the men 20% were sexually inactive before cystectomy and 35% had at least mild erection problems. These percentages increased to 50 and 91, respectively, postoperatively. Most men who tried to reach orgasm after cystectomy could do so but 36% reported that they rarely or never had orgasms. About half of the men who were orgasmic after cystectomy complained that the intensity and pleasure of the sensation were reduced. However, the desire for sexual activity did not change as drastically as the ability to function.
32 [55]	Nine women (mean age 59 years)	Radical cystectomy	Interviews	For most couples postoperative sexual activity included intercourse, although 4 women experienced severe dyspareunia and the other 3 active women had mild discomfort at first. One couple was not able to have intercourse until a session of sexual counselling 20 months postoperatively helped the wife to relax. One woman still has not been able to tolerate vaginal penetration, although she can reach orgasm through noncoital stimulation. Levels of sexual desire remained normal in 7 women but patient 9 continued to experience low sexual desire and patient 4 lost her desire for sexual activity. Of the 7 women who resumed sexual activity 6 have been able to overcome most or all of the dyspareunia that they experienced at first. The pain and discomfort included a sensation of vaginal tightness in 5 women, including 1 of the 2 whose vagina was repaired by turning the posterior wall downwards. Three women had trouble with vaginal dryness despite use of a water-based lubricant. Only 1 woman produced enough natural vaginal lubrication for comfortable intercourse.
33 (Bergman, Nilson and Peterson 1979)	Forty-two men (mean age 62.3 years)	Cystectomy, prostatectomy and vesiculcctomy	Interviews Most cases partners were also interviewed.	At the time of the interview, ten men had not attempted to resume sexual activity after the operation. Only 3 patients were able to have penile erection after cystectomy. Although the remaining 24 patients were sexually active, they did not have a normal erection after the operation. Some of the men who achieved orgasm by masturbation reported a sensation of erection during orgasm.
34 [56]	Seventy-three women (mean age 52.3 years)	Radical cystectomy with urinary diversion	Female Sexual Function Index (FSFI)	Sexual relations had ceased completely in 19 patients (26%), while it was maintained in 54 patients (74%). Among the postoperatively sexually active female various sexual dysfunctions were reported. Overall satisfaction among sexually active females was found to be the same as preoperatively in 14 patients (26%), it had worsened in 32 (59.2%) and was completely lost in eight patients (14.8%).
35 [57]	Seventy-six men (mean age of 57 years)	Cystectomy and bladder substitution	Interview	After cystectomy, only 9% of all patients were still able to have an erection every second time or more often. Thirty-eight percent stated that they were still capable of orgasm, but a rather large proportion (21%) claimed that they did not know. Of the 29 patients with retained orgasmic function, 41% reported no decrease in quality. Only 11% of conduit patients had unchanged or some coital activity post- operatively, as compared to 35% of bladder substitute patients reporting unchanged or some activity.
36 [58]	Twenty-one men (mean age 59.4 years)	Cystectomy with or without urethrectomy	Interview	All had tactile sexual activity and preserved sexual desire. Around the 12 in whom the urethra was preserved, 4 had full and long lasting erection but of short duration. These 5 patients, all had erections that was satisfactory for vaginal penetration and intercourse and four were unable to achieve an erection. Among those whom the urethra was preserved, all but three could experience orgasm. Among the nine patients treated by cystectomy and urethral excision two reported that they had weak erections insufficient for vaginal penetration and intercourse. The remaining seven failed to achieve erection. Three patients in this group experienced orgasm, one patient with a weak erection and two of the seven patients who could not achieve an erection.
37 [59]	18 pre-menopausal women (mean age of 37.8 years)	Radical cystectomy	Female Sexual Function Index (FSFI) questionnaire	Fifteen patients had an unchanged sexual life, practicing sexual intercourse at a once-weekly average rate with normal orgasms. Three patients complained of dyspareunia that was managed with treatment.

## Abbreviations

BC: Bladder cancer
BCG: Bacillus Calmette–Guérin
QoL: Quality of life
NMIBC: Non-muscle invasive bladder cancer
MIBC: Muscle invasive bladder cancer
HRQoL: Health-related quality of life
EORTC: European Organisation for Research and Treatment of Cancer
EORTC SHQ-22: European Organisation for Research and Treatment of Cancer Sexual health questionnaire - 22
TURBT: Trans urethral resection of bladder tumour
IFSF: Index of Female Sexual Function
SHIM: Sexual Health Inventory for Men
WHO: World health organisation
